# Cognitive-affective maps *extended logic*: Proposing tools to collect and analyze attitudes and belief systems

**DOI:** 10.3758/s13428-025-02699-y

**Published:** 2025-05-19

**Authors:** Julius Fenn, Florian Gouret, Michael Gorki, Lisa Reuter, Wilhelm Gros, Paul Hüttner, Andrea Kiesel

**Affiliations:** 1 Institute of Psychology, University of Freiburg, Freiburg im Breisgau, Germany; 2 Cluster of Excellence livMatS @ FIT Freiburg Center for Interactive Materials and Bioinspired Technologies, University of Freiburg, Freiburg im Breisgau, Germany

**Keywords:** Cognitive-affective mapping, Cognitive-affective maps, Network, Mixed methods, Attitudes

## Abstract

Cognitive-affective maps *extended logic* is a software package that includes three tools designed for the collection and analysis of cognitive-affective maps (CAMs). CAMs are an innovative research method used to identify, visually represent, and analyze belief systems or any semantic knowledge. By instructing participants on how to draw a CAM, they can create a visual depiction of a belief system that illustrates their attitudes, thoughts, and emotional associations regarding a specific topic. CAMs can be considered as networks enabling participants to freely draw concepts and illustrate the affective (emotional) evaluations and connections between them. To simplify the creation of CAM studies, we first developed an administrative panel for researchers which enables them to set up CAM studies without any coding. Second, to draw CAMs, a tool was developed to give participants the opportunity to create a visual depiction of their own belief system regarding a specific topic. Third, the resulting data can be analyzed using the respective data analysis app, which tracks each analysis step to make the analysis process fully transparent. As a time-efficient approach, CAMs can be used to inform exploratory research questions, like the conceptualization of surveys, or be valuable as an independent method. The tools are available under a free and open-source license. Further information, code, and comprehensive documentation are available at https://drawyourminds.de.

## Introduction

The cognitive-affective maps *extended logic* software package provides a comprehensive framework of three interrelated tools designed to visualize and analyze belief systems and, more broadly, any form of semantic knowledge. Cognitive-affective maps (CAMs) are constructed by instructing participants to depict their belief systems through interconnected concepts and to assign affective (emotional) evaluations to these drawn concepts to capture the interplay between cognition and emotion (e.g., Reuter et al., 2022; Thagard, 2010a; 2015). Crucially, incorporating affective evaluations can enhance the understanding of how emotions shape thought structures (see Section “The case for mapping affect”). The outcome of the drawing process is a network-based structure supporting both quantitative analyses, such as computing network indicators or co-occurrence matrices and qualitative approaches, including (data-driven) thematic categorization of textual content. Compared to existing methods for eliciting belief systems (*mental models*), CAMs integrate conceptual associations with affective evaluations and allow participants to freely construct and refine their belief systems without relying only on predefined categories (see Section “CAMs compared to existing tools”).

**Fig. 1:**
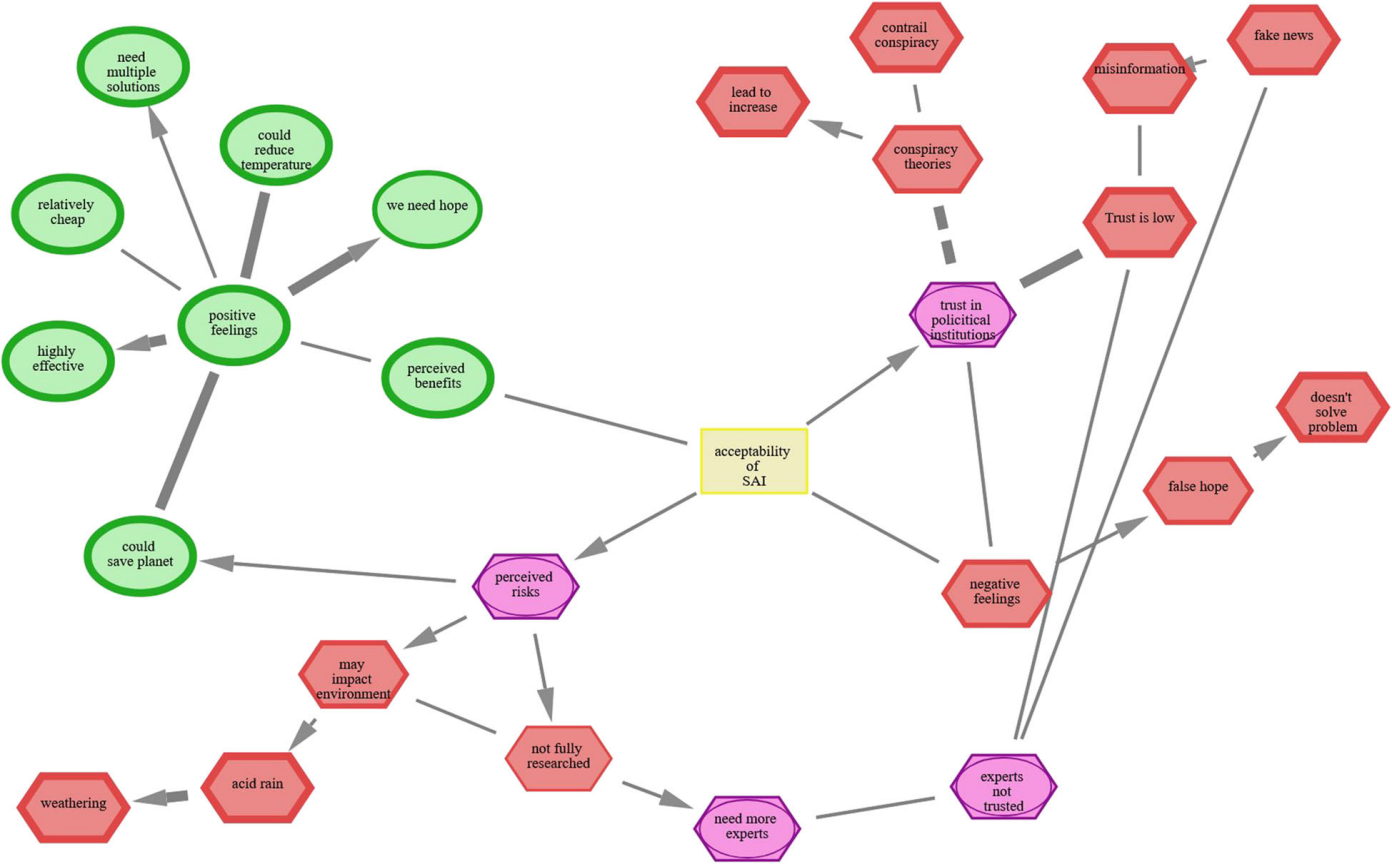
Example CAM regarding the perceived acceptability of the climate engineering stratospheric aerosol injection (SAI) drawn by a participant in the study of Fenn et al. (2023). *Note*. The concepts "positive feelings", "negative feelings", "trust in political institutions", "perceived risks", and "perceived benefits" were predefined and the concept "acceptability of SAI" was placed in the center of the CAM. Font size for concept labels has been enlarged in this example to enhance readability

Because of this flexibility, CAMs can and have been applied across diverse research domains, such as conflict resolution (e.g., Findlay & Thagard, 2014), assessment of emerging technologies (Fenn et al., 2023) or to investigate the perception of the COVID-19 pandemic (Mansell et al., 2021b; Reuter et al., 2021). Applying CAMs is feasible in various study designs, including cross-sectional assessments, longitudinal tracking of belief changes, real-time feedback in adaptive studies. Within mixed-method approaches, CAMs can also complement the design of surveys (see Section “Existing CAM research”).

### Introducing CAMs

CAMs provide a structured visualization of affective evaluations within a network of interconnected concepts on a specific topic. According to Homer-Dixon et al. (2014), they offer an immediate gestalt of the entire belief system, capturing both the simultaneous interactions between concepts and the relationships among them. For example, in Fig. 1, the CAM illustrates the perception of the acceptability of a climate technology (Fenn et al., 2023). A CAM is a network and therefore composed of vertices (referred to as concepts) and edges (referred to as connectors). Participants can freely draw concepts, which may represent goals, events, or general ideas (Thagard, 2010a). Initially, all drawn concepts are neutral (depicted as yellow rectangles). Participants can subsequently modify their affective evaluations, assigning concepts a positive (green circle), negative (red hexagon), or ambivalent (purple inscribed circle) valence (Homer-Dixon et al., 2013, 2014; Mock & Homer-Dixon, 2015). The intensity of these affective ratings ranges from -3 (strongly negative) to +3 (strongly positive) and is visually represented by the thickness of the concept’s outer border. For instance, in Fig. 1, the concept "highly effective" is rated as strongly positive. As Thagard (2010a) states, a "cognitive-affective map is a visual representation of the emotional values of a group of interconnected concepts" and thus are "conceptual structures that people use to represent important aspects of the world" (Thagard, 2010a, p. 79).

**Fig. 2:**
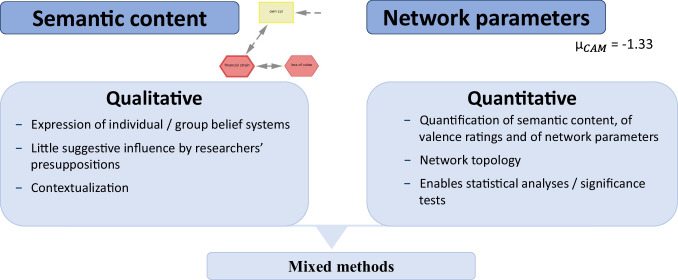
CAMs – A quantitative and qualitative research method. *Note*. At the top of the figure, the written text or comments associated with the drawn concepts represent the semantic content. The average valence of these concepts, derived from their associated ratings, serves as an exemplary quantifiable network parameter

These drawn concepts can be interconnected, with the initial connector typically indicating slight support. Connectors can be assigned varying strengths, ranging from -3 (strongly opposing) to +3 (strongly supporting), with the thickness of the connector lines visually representing the intensity of the relationship. Basically, opposing connections can be understood as a negative correlation (more A, less B), whereas supporting connections indicate a positive correlation (more A, more B; see Section “The case for mapping affect”). Such connectors can be interpreted as simple associations people have in mind, for example, in Fig. 1, the concept "highly effective" is positively associated with "positive feelings". Such a perspective is theoretically linked to word association studies (e.g., De Deyne & Storms, 2008; Slovic et al., 1991). In addition to the initial classical conception of CAMs (e.g., Thagard, 2010a; 2012a; 2014b; 2015), CAMs might contain directional arrows. These arrows indicate directional effects similar to causal relationships. For example, in Fig. 1 the participant connected "not fully researched" with a directional arrow to the neighboring concept "need more experts" indicating, that the lack of research is the cause and the need for more experts the consequence whereby the interpretation is based on the written comment that the technology "needs more research", which can also be added to concepts (for a detailed discussion of the interpretation of connections see Reuter, 2022). CAMs that contain directional arrows can thus be considered as a weighted directional network with a simple graph structure (e.g., Newman, 2018). For a more technical explanation of the data structure of CAMs, please see Appendix A.

As explained, participants can either freely draw concepts or, if researchers intend so, they can deal with predefined concepts and connections (see Section “Data collection tool”). By writing text and comments to the drawn concepts, participants elaborate further on their associations (semantic content) to the drawn concepts. Furthermore, by assigning affective ratings to each concept and by drawing connections between concepts, participants construct a quantifiable network. Therefore, as highlighted in Fig. 2, CAMs can be seen as a quantitative and qualitative research method, whereby the semantic content and network parameters can be analyzed separately or jointly (see Section “Data analysis tool” and CAM publications: Höfele et al., 2022; Livanec et al., 2022; Luthardt et al., 2020, 2022; Mansell et al., 2021a; b; Reuter et al., 2021.

#### The case for mapping affect

CAMs can be understood as a representation of *mental models*, which are inherently fuzzy, incomplete, and simplified internal representations of the external world (cf. Craik, 1943; Doyle & Ford, 1998; Johnson-Laird, 1983). These models shape cognitive and affective processes, influencing key societal and psychological phenomena, such as perceptions of climate change (Goldberg et al., 2020; Homer-Dixon et al., 2014; Sterman, 2008; Leiserowitz, 2006), the formation of conspiracy theories (Douglas et al., 2017; Uscinski et al., 2022), risk and benefit assessments of emerging climate technologies (Bellamy et al., 2016; Fenn et al., 2023; Slovic et al., 1991; Zaunbrecher et al., 2018), and responses to global crises such as the COVID-19 pandemic (Thagard, 2021; de Ridder et al., 2022; Reuter et al., 2021).

Importantly, emotions are integral to the construction and maintenance of mental models (Thagard, 2006). According to Thagard’s theory of *emotional coherence*, mental representations carry an emotional valence (positive or negative), determined by the acceptability and interconnectedness of related concepts. The valence of an element is influenced by the emotional evaluation of interconnected elements, guiding belief assimilation, modification, or resistance to change to maintain the "most coherent account of what we want to understand" (Thagard, 2000, p. 16). Consequently, CAMs are not arbitrarily generated but are structured by underlying cognitive-affective mechanisms (see Denzau & North, 1994; Johnson-Laird, 1983; Thagard, 2010b, 2012b, 2014a). This implies that individuals with similar belief systems will likely produce systematically similar CAMs when surveyed on the identical topic, e.g., due to overlapping argument structures (cf. Doyle et al., 2022; Homer-Dixon et al., 2013). Computational models such as HOTCO ("HOT COherence") simulate these interactions between cognition and emotion, demonstrating how mental coherence emerges through the interplay of reasoning, affect and motivation (Thagard, 2000, 2006; Schröder & Wolf, 2017; Wolf et al., 2015). As such, emotions do not merely accompany reasoning but actively shape cognitive processing, reinforcing coherence among beliefs, guiding decision-making and hindering or facilitating behavior.

For example, emotions influence the perception and processing of information about climate change by shaping opinion formation, truth judgments, and the acceptance of scientific findings, often independent of factual knowledge (Homer-Dixon et al., 2014; Meuer et al., 2023; Ecker et al., 2022). Consequently, emotions can both increase susceptibility to misinformation and conspiracy theories and be strategically used to make corrections more effective and promote climate-friendly behavior (Brosch, 2021; Ecker et al., 2022).

### CAMs compared to existing tools

In the context of research on mental models, different procedures to elicit mental models of participants have been discussed (Doyle et al., 2022; Jones et al., 2011; Wood et al., 2016). It is possible that the mental models are created by participants themselves (direct elicitation) or that researchers determine the structure of a participant’s mental model based mainly on textual information (indirect elicitation). For indirect elicitation, mainly structured interviews followed by a qualitative summary of the data (e.g., grounded theory or qualitative content analysis) have been applied in studies, for example, to create a “values-informed mental model” for climate risk management (Bessette et al., 2017; Mayer et al., 2017). In the case of direct elicitation, participants draw their mental model without using any software in some studies, e.g., using Post-it notes (Vink et al., 2016). In addition, multiple software solutions for direct elicitation have been developed. For example, CmapTools was developed as a graphical tool to externalize internal knowledge representations (Ifenthaler, 2010), Mental Modeller enables participants to generate system variables and connect them using weighted arrows (Gray et al., 2013) and eCASS allows to enter different types of variables and link them with unweighted arrows (Kovacs et al., 2017). More recently, M-Tool was developed to draw influence diagrams by using a predefined set of pictograms that represent system variables. These variables can be connected using weighted arrows (van den Broek et al., 2021). The pictograms in M-Tool are accompanied by audio instructions, which makes this tool extremely useful for participants with low literacy (van den Broek et al., 2021).

In contrast to M-Tool, the CAM tools presented in this article allow participants to freely add concepts to their drawn CAM. Compared to all other tools (CmapTools, eCASS, M-Tool), CAMs consider affect, which largely influences human thinking (see Section “The case for mapping affect”). Further based on the computer program EMPATHICA (Thagard, 2010a), a now unsupported software called Valence (Rhea et al., 2020) was developed to draw CAMs and applied in a previous dissertation project by Reuter (2022). The data collection tool presented in this article (see “Data collection tool” section) extends the functionalities of Valence, by offering more flexible study configurations, such as allowing researchers to modify study parameters (e.g., the number of concepts, types of connections, and task constraints) based on the specific research design. This flexibility was enabled by providing a strong data model (see “Appendix A Technical details, CAM parameters” section).

### Existing CAM research

*The most up-to-date information on CAM research is available in the official online reference. This resource provides comprehensive insights into the programmed tools, as outlined in the subsequent sections and includes a step-by-step explanation on how to set up studies. For further details, please visit the project homepage at* https://drawyourminds.de/.

**Table 1:** List of possible key applications of CAMs

Research stage	Study design	Application
Exploratory phase	single-time elicitation	Capture the cognitive-affective representation an individual or group holds regarding a specific topic at a single point in time (e.g., to inform the main research question)
Pre-study phase	Mixed-method design	Integrate CAMs into mixed-method research designs to complement questionnaires, allowing for free association beyond pre-specified questionnaire scales (e.g., to inform main study design)
Main study	Pre-post intervention designs	CAMs serve as a dependent variable where participants create two distinct CAMs before and after an intervention or modify an initial CAM at a subsequent time point to reflect changes (e.g., to test intervention)
Main study	intervention design	Expose participants to a belief system that is not their own, assessing the impact of viewing another’s cognitive-affective representation on their perspectives (e.g., for conflict mediation)
Main study	Adaptive designs	Based on real-time analysis of the CAMs drawn by participants, specific feedback questions, for example, regarding their overall affective evaluation can be administered

*Note*. For updated possible key applications of CAMs please read the "Set up study" section in the online documentation

The relevance of CAMs as a research method in its own right lies in the method’s multiple fields of application. In conceptual/qualitative studies, CAMs have been applied to depict political ideologies (e.g., Clapp, 2021; Homer-Dixon et al., 2013; Thagard, 2015), to support conflict resolution (e.g., Findlay & Thagard, 2014; Homer-Dixon et al., 2014; Mock & Homer-Dixon, 2015; Thagard, 2018, 2021), or to inform international climate politics (Milkoreit, 2017). In quantitative studies, CAMs were used to investigate the perception of the COVID-19 pandemic (Mansell et al., 2021b; Reuter et al., 2021), to evaluate the support of a carbon tax (Mansell et al., 2021a), or to identify ethical principles of students (Höfele et al., 2022). Using a mix of qualitative and quantitative procedures, CAMs have been applied to assess the perception of emerging technologies (Fenn et al., 2023) or the success of an intervention in early child-care institutions (Luthardt et al., 2020, 2022). CAMs have also been used in the context of agent-based modeling (Schröder & Wolf, 2017; Wolf et al., 2015) by implementing the multiple constraint satisfaction process proposed in Thagard’s hot cognition model (cf., Thagard, 2006; 2018). As can be seen in the multiple publications, CAMs can be employed at various stages of the research process. Table 1 outlines several key applications of CAMs, highlighting the broad spectrum of opportunities CAMs offer in research.

Multiple other applications are possible: As explained in Reuter et al. (2022), CAMs are related to semantic networks because knowledge of participants is represented as a network-like graph (e.g., Lehmann, 1992). Further, if CAM studies contain arrows (directional influences), CAMs are related to fuzzy cognitive maps since some kind of causal/ associative relation is assumed between such related concepts (e.g., Kosko, 1986). As such, CAMs could also be applied in the context of word association studies (e.g., De Deyne et al., 2019; Leiserowitz, 2006) or for creating knowledge graphs (e.g., Tripto et al., 2016).

**Fig. 3:**
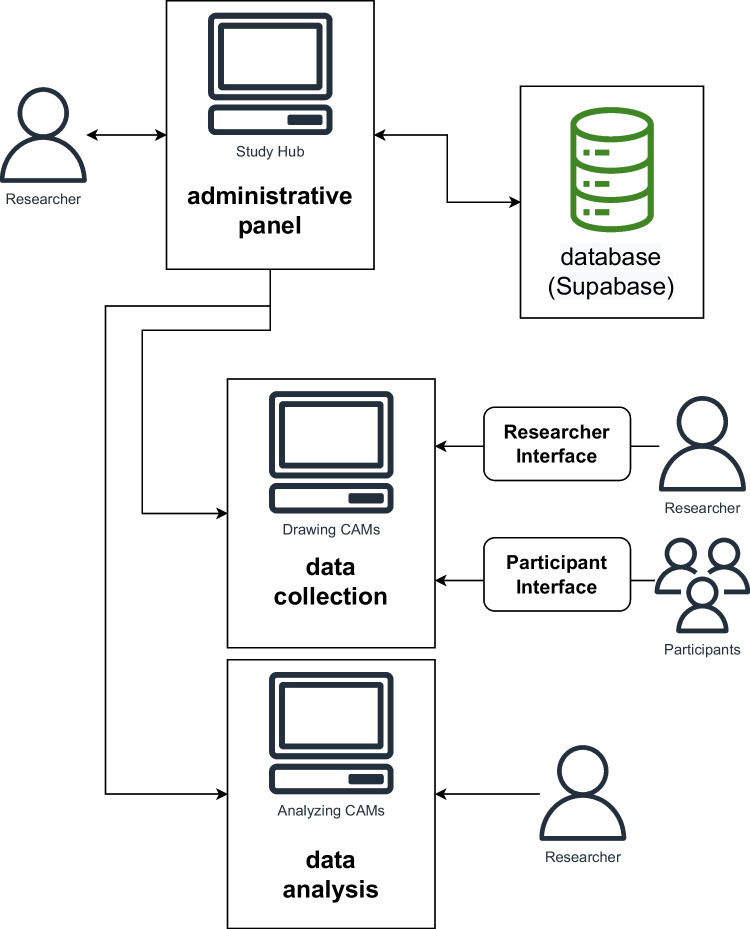
Overview of the developed CAM tools. *Note*. Researchers can set up studies via the administrative panel and configure them through the researcher interface of the data collection tool. The collected data can subsequently be analyzed using the data analysis tool. Drawn CAMs can be collected on our integrated Supabase server

## Cognitive-affective maps *extended logic*

The cognitive-affective maps *extended logic* software package is based on a previous dissertation project by Reuter (2022), which showed for the first time that large online CAM studies are feasible using the Valence software (Rhea et al., 2020). The main goal in developing this software package is to enable establishing gold standards – especially for the data model and analysis pipelines. To address this, we created three interconnected tools: First, to simplify the creation and management of CAM studies, we designed an administrative panel for researchers. Second, we developed a sophisticated and adaptable data collection tool that allows participants to visually depict their belief system of specific topics. Finally, we introduced a data analysis application that meticulously tracks each step of the analysis process to ensure full transparency. An overview of these tools and their interrelationships is provided in Fig. 3. All the tools are available for free as open-source software under the MIT License and can be downloaded from our GitHub repository (https://github.com/CAM-E-L). Both the data collection tool and the data analysis tool can be used offline and are thus suitable in contexts where an Internet connection is not available or not desirable (e.g., for reasons of data security). To adhere to the best standards of research (Reips, 2021; Sauter et al., 2020) and ensure high data quality, the three tools have been rigorously tested^1^ and already applied in some studies^2^.

The individually developed tools are described in more detail below, whereby technical specifics (such as programming languages used) are explained in Appendix A. We recommend interested readers who want to test our tools hands-on to visit our administrative panel.

### Administrative panel

The administrative panel (https://drawyourminds.de/) and the online documentation (https://osf.io/q5hj4/) provide central information on the tools and about the process of creating and analyzing a CAM study (see also “Guidelines” section for recommendations). Using this panel, researchers can easily include CAMs in online studies, eliminating the need for researchers to set up a server themselves. Researchers can register, log in, and create a study. Through the interface, several adjustments of the study design are possible (see “Data collection tool” section). Upon adding the study, the panel generates a link to share with participants and redirects the participants after they have drawn their CAM (see “Guidelines” section).

For the single studies, real-time descriptive statistics (e.g., participant count, date of last CAM collected, average affective evaluation) are provided, and the CAM data of each participant can be downloaded for data analyses (see “Data analysis tool” section).

### Data collection tool

**Fig. 4:**
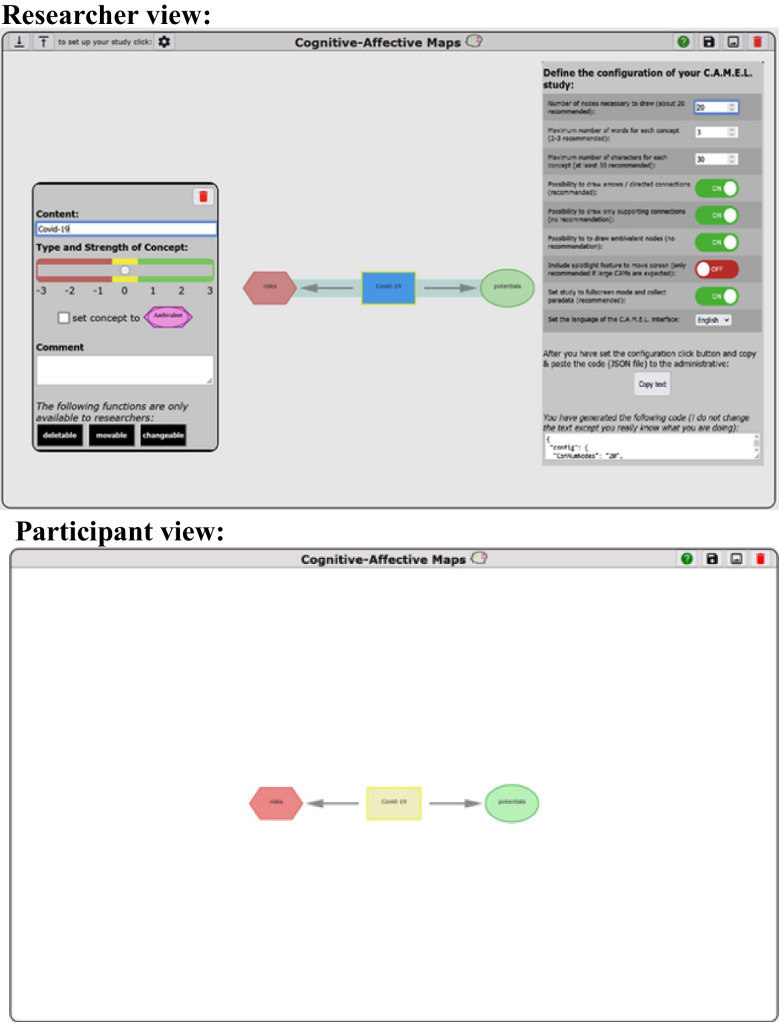
Two different interfaces of the data collection tool. *Note*. The researcher interface enables researchers to set up and configure CAM studies and the participant interface enables participants to draw a CAM, whereby the related CAM data are stored for later data analysis

The data collection tool is designed to configure CAM studies and collect CAMs. It offers a researcher interface in which researchers can set up studies, and a participant interface in which participants can draw CAMs (see Fig. 4).

**Table 2:** Parameters which can be individually changed using the researcher’s view of the data collection tool

Parameter	Meaning	Possible values
#MinNumNodes	Number of concepts needed to be drawn before the participant can save the CAM.	1–50$^{1}$
#MaxNumWords	Maximum number of words allowed for each concept.	1–5$^{2}$
#MaxLengthChars	Maximum number of characters allowed for each concept.	30–300
#enableAmbivalent	If ON, it is possible to draw ambivalent concepts.	ON, OFF
#enableArrows	If ON, it is possible to draw arrows/ directed connections between concepts.	ON, OFF
#BidirectionalDefault	As default, the drawn connection is bidirectional, only makes sense if #enableArrows is set to ON.	ON, OFF
#OnlyStraightCon	If ON, only supporting/ straight connections can be drawn.	ON, OFF
#cameraFeature	If ON, a spotlight feature is included to move the drawing screen. If participants move their mouse to the edges, the drawing screen is moved to the respective side.	ON, OFF
#fullScreen	If ON, the study is set to fullscreen mode and paradata is collected (defocus, focus events).	ON, OFF
#setLanguage	Set the language of the interface of the data collection tool.	English, German, Spanish, Chinese

*Note*. $^{1}$Maximum number is restricted, because the drawing space is limited. $^{2}$It is highly recommended to set this value to 1–3 if you are aiming to summarize/ aggregate the CAM data. Instruct participants to avoid writing sentences and to draw multiple concepts instead

#### Researcher interface

Researchers use this interface to set specific parameters for the participant interface and to provide an initial CAM with which participants start. An overview of possible parameters which can be adjusted depending on the research question of the respective CAM study is given in Table 2. The researcher can set specific requirements regarding the minimum number of concepts that each participant has to draw before the CAM can be saved. Further, the maximum number of words per concept as well as the maximum number of characters per CAM can be specified. Regarding the affective assessment per concept, the researcher can choose to disable ambivalent assessments. Regarding the connections between concepts, the default mode enables solid and dashed lines, that is supporting and opposing connections. Here, the researcher can decide to enable only solid (supporting) connections and whether connections can be unidirectional, e.g., drawing arrows instead of lines. In case that arrows are enabled, the researcher can chose whether a connection is initially drawn bidirectional and then has to be changed to unidirectional arrows by the participant. In addition, the researcher sets parameters regarding the global configuration of the study. Here, researchers set the language of the study and whether the window of the data collection tool is set to fullscreen mode (in this mode also paradata regarding defocus events is collected). Further, a spotlight feature might be switched on, which enables participants to move the drawing screen while creating their CAM.

While researchers can provide a blank surface for participants to create their CAM (e.g., Mansell et al., 2021b), they could also predefine an initial CAM with specific concepts (e.g., Fenn et al., 2023; Mansell et al., 2021a; Reuter et al., 2021). Such an initial CAM can contain one central concept mentioning the topic of the CAM, contain two opposing concepts or even might present a full CAM and ask participants to change it according to their own belief system. These predefined concepts can be set as non-deletable, immovable and its text unchangeable by participants. A comprehensive discussion on the implications of study design, including the impact of predefined concepts and possible parameter settings (outlined in Table 2), is provided in the “Guidelines” section.

**Fig. 5:**
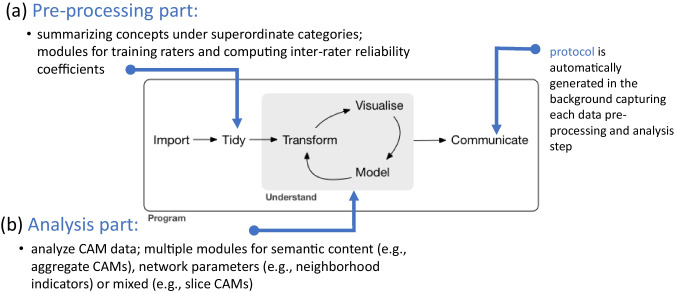
Data analysis pipeline of the data analysis tool. *Note*. The data analysis tool consists of a pre-processing step for data summarization (**a**) and an analysis step (**b**). A detailed flow chart illustrating the individual implemented modules is available in the ”Data analysis tool” section of the online documentation under the subsection "Implemented features overview". Picture from Wickham, H., & Grolemund, G. (2017). R for Data Science: Import, Tidy, Transform, Visualize, and Model Data. O’Reilly Media, Inc

#### Participant interface

This interface offers participants an easy and intuitive way to draw their CAM. The entire logic of the study runs on the client side and whenever a participant interacts with the data collection tool, the central CAM object is updated in real-time. Such an approach of saving data temporarily on the client side (i.e., the computer used by the participant), eliminates network latency (cf., Henninger et al., 2022). When a participant clicks on the save button, the data collection tool checks whether the participant has drawn the defined minimum number of concepts (#MinNumNodes parameter in Table 2) and whether there are concepts that are disconnected from the CAM. If all conditions are fulfilled, the data representing the CAM is sent to the server.

The structure in which the data is saved follows a common data structure for network analysis and therefore can be used in programmed frameworks for network analysis like igraph (Csardi & Nepusz, 2006). Thus, researchers who want to develop their own innovative methods for analyzing CAMs or who want to apply analytical methods from other fields (e.g., Briganti et al., 2022; Talaga & Nowak, 2022) are enabled to do so. For a detailed description of the data structure of CAMs, see Appendix A.

### Data analysis tool

The data analysis tool can read CAM data generated with the data collection tool or with the Valence software (Rhea et al., 2020), which was developed as a follow-up to EMPATHICA (Thagard, 2010a) and used for previous CAM studies (see homepage of Paul Thagard for a list of CAM publications^3^). Programmed in Shiny, an R package that enables the creation of interactive web applications (Wickham, 2021), researchers can easily pre-process and analyze their collected data. Applying the logic of modular programming (e.g., Seydnejad, 2016), the application is currently broken down into 28 specialized modules applying 34 functions written in R and Python. The logic of the data analysis tool follows the principle of a classical data-analysis pipeline (e.g., Peng & Matsui, 2016; Wickham & Grolemund, 2017), whereby the use of the application is composed of two steps (see Fig. 5): (a) in the *pre-processing part*, it is possible to summarize the CAM data computer-assisted (e.g., by use of sophisticated language models). The CAM data can be summarized by multiple collaborators and the reliability (inter-rater consensus) of the process can be quantified and increased using the implemented reliability module. (b) In the *analysis part*, the pre-processed data can be analyzed and visualized using multiple implemented functions (e.g., aggregate the individual drawn CAMs). An overview of the functions of the data analysis tool separately for the pre-processing and analysis part is given in Tables 3 and 4. In the following, we briefly describe the implemented functionalities^4^.

The data analysis tool guides the researcher through the analysis process. Before applying any pre-processing or analysis functionalities, the researcher needs to upload the raw CAM data and optionally (if already existent) a previously generated analyses protocol. Whenever a researcher interacts with the data analysis tool, a protocol is automatically generated in the background, capturing each data pre-processing and analysis step. After uploading the protocol in the data analysis tool, multiple protocol statistics (e.g., which summarizing functionalities have been used) can be inspected. This protocol helps to make the summary process of the CAM data fully transparent for other researchers. In line with Tuval-Mashiach (2017), we think it is of great importance to document and track all steps in the pre-processing and analysis process to increase transparency in qualitative as well as in quantitative research (see also Moravcsik, 2014). As we will show in the “Guidelines” section, the process of summarizing drawn concepts in CAMs to superordinate concepts has some degrees of freedom, whereby we propose a five-step procedure to summarize CAM data (cf., Fenn et al., 2023). After uploading the raw CAM data (and optionally the protocol), each CAM is graphically depicted, enabling the researcher to get an overview of the data set. In this module, it is possible to sort the participants’ CAMs according to different criteria (e.g., number of drawn concepts) and delete single CAMs (e.g., CAM drawn about wrong topic). This step prepares the data set for further processing.

**Table 3:** Pre-processing functionalities of the data analysis tool

Modules	Function	Purpose
To summarize concepts under superordinate categories	Approximate Matching	Generating suggestions for summarizing concepts under a superordinate concept. By using approximate string matching, string distances between all unique concepts in the dataset can be computed (using optimal string alignment) to find words which have been written slightly differently.
	Searching terms	Generating suggestions for summarizing concepts under a superordinate concept. Search concepts, using regular expressions in CAMs for specific terms that were mentioned, summarize some or all of these concepts.
	Search for synonyms	Generating suggestions for summarizing concepts under a superordinate concept by automatically searching for synonyms in a dictionary.
	Apply word2vec Model	Generating suggestions for clustering and summarizing concepts according to the cosine similarity between (single-word) concepts. Cosine similarity is computed using pre-trained large language models from the Python library spaCy.
	Overview of non-summarized concepts	Gives an overview of all concepts/terms that have not been summarized under a superordinate concept, yet.
To investigate inter-rater reliability	Train raters for summarizing of concepts	Helps to instruct raters and to draw random subsets of unique concepts of the data set on which to train raters.
	Compute inter-rater reliability	Computes the inter-rater reliability for summarizing concepts. Assess if raters tend to summarize the same concepts together under one superordinate concept, regardless of the exact name they allocate to the superordinate term.

*Note*. Detailed information on the functionalities of the modules is available in the online documentation in the ”Data analysis tool” section, see https://camtools-documentation.readthedocs.io/en/master/Data%20Analysis%20Tool/

**Table 4:** Analysis functionalities of the data analysis tool

Modules	Function	Purpose
To compute network indicators	Network indicators	Compute several network indicators (e.g., mean valence, density etc.) on an overall CAM level (macro). Additionally, select one or several concepts and calculate network indicators on an individual concept level (micro).
	Neighborhood indicators	Compute mean valences over neighborhoods of concepts of order 1/2 with adjustments for positive concepts and weighting (in total six variants).
	Summary statistics	Get a descriptive summary of network statistics you have calculated, get an APA-formatted table of statistics, get a correlation plot between different network indicators and search for significant correlations between network indicators.
To get outputs for concepts on word level	Create word list	Create a word list with summary statistics for every concept (mean/ SD valence, mean/ SD degree).
	Create word cloud	Create a word cloud of all the concepts in the dataset with colors according to the word’s mean valence.
	Graphics and statistics	Create a pie chart, barplot (APA 7 format), and table for every summarized superordinate concept in your dataset separately.
	Get summary statistics for all concepts	Get a table containing all unique summarized concepts and their respective frequencies (separated by *N* = total, $N_{positive}$ = positive, and so on) separately for each CAM.
To aggregate CAMs	Aggregate CAMs	By creating a so-called “canonical adjacency matrix”, CAMs are aggregated according to different criteria (all CAMs, CAMs of a certain group), whereby the size of the concept and the thickness of the connection is proportional to the frequency of the drawn concepts and the pairwise connections respectively.
To cluster CAMs on concept level	Concept co-occurrences	Computing the concept-co-occurrences between all CAMs by setting up multiple contingency tables, followed by computing the phi coefficient, groups of concepts with similar concept-co-occurrences in CAMs are identified.
	Valence co-occurrences	Computing hierarchical clustering over the given valence ratings over all concepts assigned to the same superordinate category of the data set to identify similar CAMs.
To slice CAMs	Slice CAMs	If you have a CAM structure which can be separated (e.g., predefined opposing concepts), the CAMs can be automatically sliced according to two possible criteria: (a) delete a connection between two concepts, and/ or (b) delete a concept. Automatically, the CAM is changed that way and checked according to multiple criteria (e.g., the number of expected network components) to validate the slicing process.
	Summary statistics	Get summary statistics (e.g., an APA-formatted table of statistics and within *t* tests) for the so-sliced CAMs.
To get a report for the articles	Get report	Get a report in APA 7 format with multiple descriptive statistics, which could be copied in an article or sent to other stakeholders. Additionally, it is possible to get summary statistics for individual concepts.

*Note*. Detailed information of the functionalities of the modules are written in the online documentation in the ”Data analysis tool” section, see https://camtools-documentation.readthedocs.io/en/master/Data%20Analysis%20Tool/. Remark: Degree is the number of connections incident to a particular concept in a CAM, while we do not differentiate between in-degree and out-degree here

**Fig. 6:**
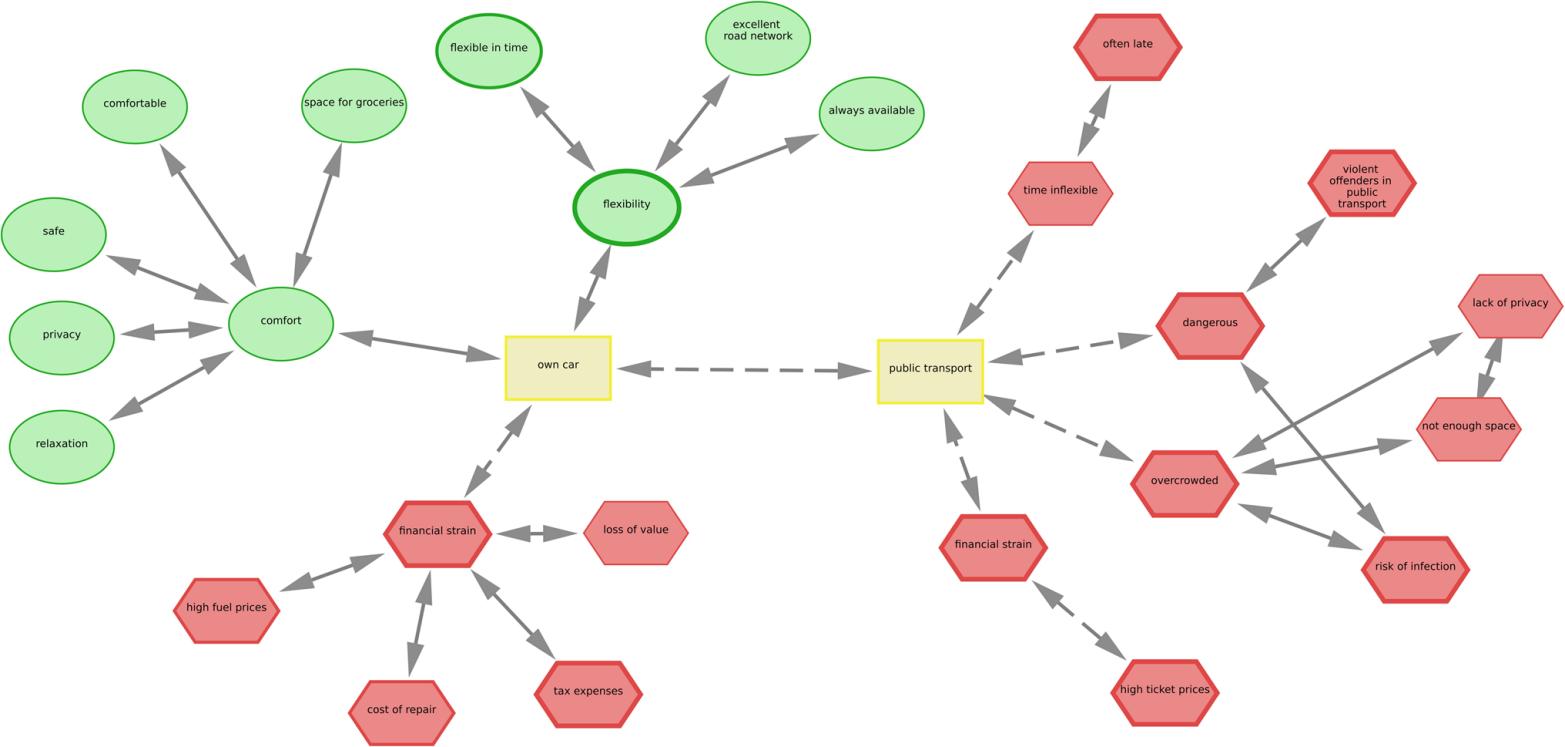
Participants indicate their motivation to use their own car or public transport. *Note*. CAM was drawn by a participant in the study by Sendtner (2021). In the most recent version of the data collection tool, bidirectional connections do not contain any arrow heads

#### Pre-processing part

Pre-processing functions refer to modules for summarizing concepts into superordinate categories and to modules for training and computing inter-rater reliability. As shown in Table 3, *four different approaches are possible to summarize CAMs*. The "Approximate Matching" function using the stringdist package in R (van der Loo, 2014) applies the optimal string alignment distance to compute the distances between two strings. For example, using this method, the distance between "dreams" and "dreasm" (spelling error) would be d = 1, because the adjacent character "s" would be transposed. Such a procedure to correct for spelling errors was already proposed by Damerau (1964).

The second function "Searching terms" uses so-called regular expressions, which is a concise syntax to describe patterns of text (Friedl, 2006). Here we apply the stringr package, which is part of tidyverse (Wickham et al., 2019). The stringr package allows to search for similar terms, e.g., by using the expression "[[:digit:]]", all drawn concepts including any digits can be identified^5^.

The next two functions enable the researcher to automatically summarize concepts based on semantic similarity^6^. The function "Search for synonyms" uses the English synonym dictionary included in the qdap R package (Rinker, 2020), in which all synonyms for single-worded concepts are automatically searched. If there is an overlap between found synonyms, a list of overlapping synonyms is suggested for summary. For example, the concepts "war" and "conflict" would be identified as synonyms. The function "Apply word2vec Model" is based on pre-trained language models from the Python library spaCy (models are trained by the deep learning library thinc, see Honnibal et al., 2021). By comparing word vectors, it is possible to compute the cosine similarity between drawn concepts pairwise to identify groups of drawn concepts with similar meaning (e.g., Mikolov et al., 2013; Srinivasa-Desikan, 2018). For example, using the largest currently available pre-trained English language model (“en_core_web_lg”)^7^, cosine similarity between the words "responsibility" and "accountability" would be 0.70. Cosine similarity ranges from -1 (indicating opposite vectors) to 1 (indicating proportional vectors). Researchers can set a threshold for cosine similarity to decide when concepts are similar enough to be summarized together. This threshold allows them to control which suggested summarizations to accept or reject. Finally, the "Overview of Non-Summarized Concepts" function provides a list of all concepts that have not yet been summarized into superordinate concepts, allowing researchers to review and address any outstanding concepts.

To *assess inter-rater reliability*, CAM data might be summarized by several independent raters. For such a case, we implemented a *reliability module* (see last two rows in Table 3), which was motivated by literature on inter-rater reliability (Gisev et al., 2013; Hallgren, 2012; ten Hove et al., 2018). In the sub-module "Train reliability", a list randomly drawn from all unique concepts of the data set (e.g., 10% of all unique drawn concepts) is generated in .xlsx format. This file can be downloaded and sent to the raters together with the proposed instructions. After the raters have summarized all concepts from the list to superordinate categories, the separate coding files of all reviewers can be uploaded and reliability coefficients can be computed in the "Get Reliability" sub-module. Please note that the reliability coefficients only depend on the assumption that the same groups of concepts are categorized to a specific superordinate category each by different raters, but not that identical terms are used for the respective superordinate categories by each rater.

Three possible reliability coefficients can be computed: (a) Cohen’s kappa is computed pairwise between all raters by assuming a perfect match of overlapping categories for each concept, (b) Cohen’s kappa is computed pairwise by maximizing overlapping concepts, which have been summarized, and finally (c) Fleiss’ kappa and category-wise kappa (Fleiss et al., 2013) for different groups of categories is computed. Additionally to these reliability statistics, summary statistics of the group of concepts assigned to superordinate categories are given. This information can be used to train raters, who could subsequently summarize the complete CAM dataset.

#### Analysis part

As shown in Table 4, we sorted the functions for CAM data analyses into six modules. All implemented functions were motivated by graph theory/ network analysis (e.g., Diestel, 2017; Newman, 2018), multilayer networks (e.g., Bianconi, 2018; Domenico, 2022), semantic networks (e.g., Bernard et al., 2016; Borge-Holthoefer & Arenas, 2010), content analysis (e.g., Mayring, 2022; Prior, 2014), and empirical CAM articles (especially Luthardt et al., 2022; Mansell et al., 2021a, b).

The module *network indicators* is based on the igraph package (Csardi & Nepusz, 2006) and enables researchers to currently compute 33 different network indicators which are described in detail in the online documentation. The indicators can be computed on a micro-level, which extracts features on the single concept level, on a mezzo level, which extracts features of meaningful subgraphs (called communities), and on a macro level, whereby the whole CAM is considered (cf., Soundarajan et al., 2014). It is also possible to compute so-called *neighborhood indicators*, whereby different mean valence scores are computed for groups of concepts. This is helpful if researchers have specified two contradictory concepts in a study as a starting point in order to analyze the attitudes and belief systems referring to two opposing concepts. As an example, in Fig. 6 participants were tasked to indicate their attitudes toward the use of their "own car" or "public transport" during the COVID-19 pandemic. For this CAM, it is not meaningful to compute the overall mean valence. Instead, the mean valence over the neighborhood of the respective concept of the first or second order (one or two steps away, i.e., concepts that are directly connected to the respective concept) indicates the negative assessment of "public transport" and the mixed assessment of "own car". In the data analysis tool, it is also possible to temporarily remove connections (and/ or concepts) in order to not distort the neighborhood statistics (e.g., mean valence two steps away from the concept "own car"). See also module "Slice CAMs" below for more options to analyze data for such study designs.

Please note that many of these network indicators have not yet been used for data analysis in CAM studies and therefore are not yet validated. Our aim here is to provide a set of diverse indicators so that future research on CAM studies can easily check whether these indicators are useful and explore whether they turn out as valid measurements. Up to now, the network indicators mean valence, central node valence, density, diameter, number of nodes, number of links, percentage of negative, positive, and ambivalent concepts were significant predictors in different studies (see Mansell et al., 2021b; Reuter et al., 2021)

The following modules, except the "Get report module", enable summarizing CAMs on a semantic level (written texts by participants). On an overall concept level, it is possible to create a *word list* in .xlsx format with summary statistics for each concept ($mean_{valence}$/ $SD_{valence}$, $mean_{degree}$/ $SD_{degree}$). Multiple options are possible like creating a word list of the drawn raw concepts or the summarized concepts, or split the summarized words by their respective valence (e.g., "Cost_positive" vs. "Cost_negative") to investigate the different meanings people associate with identically named concepts. Applying the wordcloud package (Fellows, 2018), *word clouds* can be drawn, whereby the colors indicate the concept’s mean valence. On a single concept level, a *pie chart, barplot, and table for each summarized superordinate concept* can be created to reflect and discuss the summary process of the CAM data. For all single, unique summarized concepts, it is possible to create an *file in .xlsx format containing the respective frequencies of the drawn concepts separately for each CAM* to easily analyze, for example, how often a certain concept with a specific valence was drawn within a CAM dataset.

The module to *aggregate CAMs* offers different options: (a) aggregate a number of randomly chosen CAMs, (b) aggregate the most positive/ negative CAMs regarding mean valence, and (c) choose CAMs with specific IDs you want to aggregate. The aggregation process involves creating a "canonical adjacency matrix" (motivated by Luthardt et al., 2022; Prior, 2014). This matrix is visualized as a static and a dynamic network using the igraph package (Csardi & Nepusz, 2006) and the visNetwork package (Almende et al., 2021) to provide an overall graphical representation for any number of CAMs (see Fig. 13 in “Appendix B Application example” section). The size of each concept and the thickness of the connections are depicted proportional to the frequency of the drawn concepts and connections, respectively. Internally, the overall graphical presentation is realized by renaming the concept with identical terms in the individual CAMs. For example, if in a single CAM the concept "cost" is written twice, these two concepts would be renamed to "cost_1", "cost_2", whereby the concepts are named in descending order of their degree.

The *co-occurrences of concepts* within individual CAMs is computed by setting up multiple contingency tables between single pairs of summarized concepts. Imagine a CAM dataset in which multiple concepts were summarized to the superordinate concepts "do something" and "accountability" (see Fenn et al., 2023). In total, the concept "do something" was drawn 18 times (after summarizing) and the concept "accountability" 13 times. Seven participants drew both concepts together in their respective CAM. This resulted in a significant phi coefficient ($\phi =.27$, $p <.05$), indicating that both concepts were drawn together disproportionately often. This computation is repeated pairwise for all concepts and the pairwise phi coefficients are visualized within a heat map. In the online documentation, we explain how we adjusted this procedure to also account for the valence of the summarized concepts. On the conceptual level, it is also possible to compute the *valence co-occurrences*, whereby the mean valence over all summarized concepts is computed. These mean variables are *z*-transformed and a hierarchical cluster analysis (Euclidean distance and Ward’s method) for all summarized concepts which were drawn at least two times is applied. The resulting cluster solution can be interpreted based on the average mean differences in the mean variables of the summarized concepts. This analysis might help to identify if identically named/ summarized concepts of certain supporters/ opponents (e.g., on belief in climate change) have different affective ratings. This approach is inspired by the work of Homer-Dixon et al. (2014), who suggested that if emotions significantly influence the severity and persistence of conflicts, it may be more effective for disputants or mediators to focus on altering the affective evaluation associated with concepts rather than attempting to change the concepts themselves.

The module to *slice CAMs* automatically separates CAMs according to two possible criteria: (a) delete a connection between two concepts, and/ or (b) delete a concept. For example, in Fig. 6, by deleting the connection between the two opposing concepts "own car" and "public transport", the CAM could be separated into two "sub-CAMs" (called components). The slicing process is checked according to multiple criteria (e.g., number of expected network components is two) to validate the slicing process. The so-generated datasets could be uploaded again to the data analysis tool in case the resulting sub-CAMs (e.g., to investigate reasons to use the "own car") should be summarized separately.

To simplify the reporting of CAM studies, a module to *get a report in APA 7 format* was developed. Using the module, a description of the CAM dataset, statistics of the summary process (pre-processing part of the data analysis tool), and (if desired) statistics of individual concepts are generated. This report could be copied to a scientific publication or sent to interested collaborators.

**Fig. 7:**
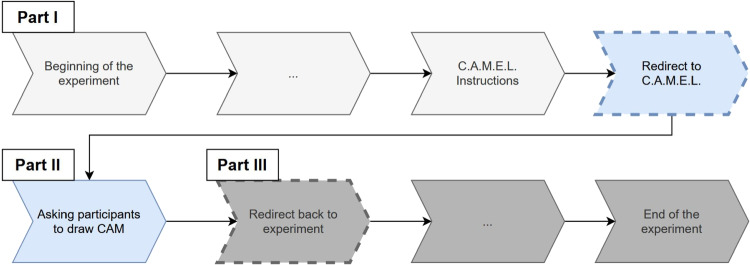
Three parts of a self-administered online CAM study. *Note*. The *dashed lines* indicate a redirect between different parts/servers

## Guidelines

In the following section, we will provide recommendations on how a researcher can set up a CAM study, highlight the influence of different study designs, and explain how to summarize CAM data. After reading the guidelines section, we encourage the interested reader to explore the “Appendix B Application example” section. Here we present the analysis procedure for a data set where participants reflected on their thoughts and feelings during the COVID-19 pandemic. Additionally, we recommend hands-on testing our tools via the administrative panel. More comprehensive and updated information is available in the online documentation.

### Setting up CAM studies

As shown in Fig. 7, an online CAM study typically consists of three parts (cf., Fenn et al., 2023; Mansell et al., 2021a, b; Reuter et al., 2021): (1) a context is set up for the participants (e.g., welcome screen, informed consent) and participants receive instructions on CAMs in general and how to use the data collection tool to draw CAMs^8^, (2) participants draw their CAM, and finally (3) participants answer follow-up questions.

Part (1) and part (3) can be set up using well-known online frameworks like SoSci Survey (Leiner, 2023), oTree (Chen et al., 2016) or lab.js (Henninger et al., 2022). For part (2), the data collection tool can be configured using the described administrative panel (or hosted and configured by the researcher on their own server^9^). The administrative panel returns a link, which can be integrated in part (1) and can link to part (3), whereby the collected data can be matched via URL parameters^10^. For a detailed description on how to set up an online CAM study, please read the ”Set up study” section in the online documentation.

### Possible study designs and their influence on CAM data

When participants create CAMs through direct elicitation, the process of *knowledge elicitation*, which involves systematically gathering and converting tacit mental models into explicit, analyzable formats, is crucial for ensuring the accuracy and relevance of the resulting maps (Hodgkinson & Clarkson, 2005). Using our developed Data Collection Tool, task constraints can be introduced (a) through the specification of software parameters, such as the minimum number of concepts required for each CAM, or if only supporting bidirectional connections are allowed; and (b) by predefining concepts. The defined software parameters, along with the definition of predefined concepts, directly influence the structure and complexity of the resulting CAMs. Specifically, free association tasks, where participants are allowed to generate concepts independently, could promote an ideographic approach that captures individual-specific mental models. In contrast, recognition tasks, where participants are limited to predefined concepts, could align with a nomothetic approach and produce highly standardized and comparable CAMs (cf. Doyle et al., 2022; Romolini et al., 2012; Ruiz-Primo & Shavelson, 1996). Predefining concepts, as seen in Fenn et al. (2023), can introduce bias by priming participants to focus on specific concepts, potentially limiting the diversity of their responses, but also facilitating the semi-automated summarization of the data.

**Fig. 8:**
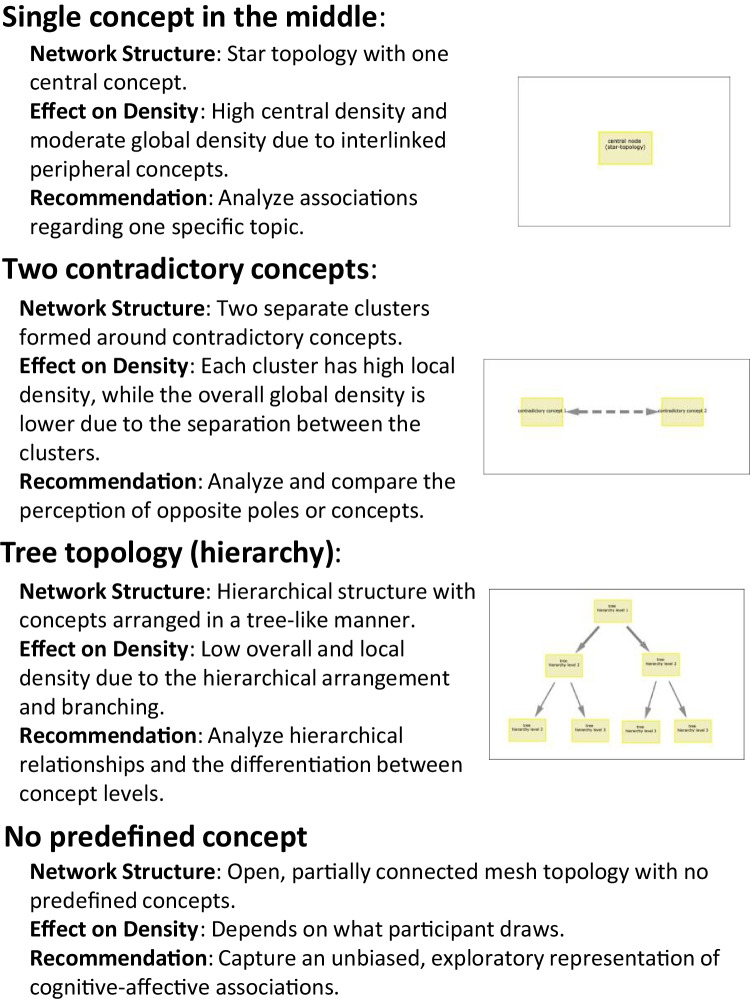
Possible arrangement of predefined concepts (network topology) and recommendations. *Note*. Network topology in this context refers to the arrangement and interconnection pattern of concepts within a CAM, defining its structure and layout

**Fig. 13:**
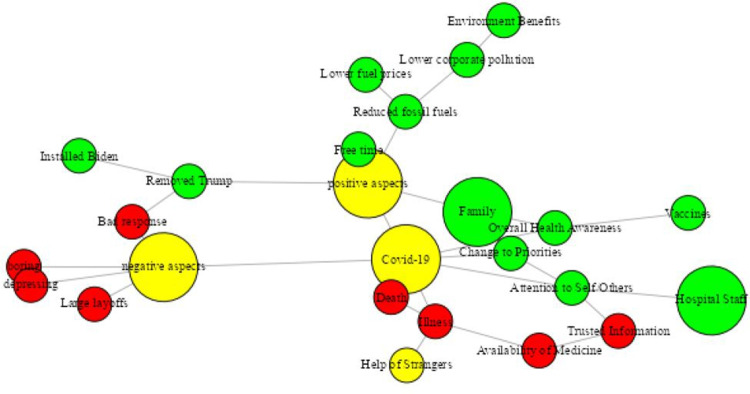
Two most positive CAMs in the dataset aggregated. *Note*. In the visual representation, the *yellow* is indicative of neutral concepts, *green* is used for positive concepts, and *red* is for negative concepts. The classification of a concept as neutral is based on the criterion that its mean valence falls within the range of [-0.5, 0.5]. This range is chosen to signify the presence of mixed emotional sentiments associated with concepts. The size of the concepts and the connections is relative to their frequency

Further based on our previous studies, we found out that the arrangement of predefined concepts and connections as starting points for participants affects the data generation process (discussed for the first time in Kreil, 2018). For example, a single central concept typically results in a network resembling a star topology, where many concepts are connected to the central concept (Mansell et al., 2021b; Reuter et al., 2021; Gros et al., 2021). Starting with two contradictory concepts leads to two clusters of drawn concepts around the respective opposites (Sendtner, 2021). Additional variants of how to arrange predefined concepts are viable. For example, defining a tree topology with concepts at the top of the drawing interface encourages participants to "add leaves to a predefined tree" in a hierarchical manner, thereby differentiating and diversifying the initial network (unpublished data). The absence of predefined concepts can result in a partially connected mesh topology, where many concepts are interconnected (e.g., Mansell et al., 2021b). In Fig. 8, based on both existing and unpublished CAM data, the effect on the resulting CAM topology and the density of the drawn connections is illustrated, along with recommendations on the suitability of each setup of predefined concepts. The effect on density refers to the concentration and distribution of drawn connections within the network, which can vary depending on the structure of predefined concepts, such as whether they promote clustering or more evenly spread connections across the network.

Finally, increasing task constraints through software parameters (e.g., limiting connections to bidirectional ones and excluding ambivalent concepts) significantly reduces study duration. Researchers should consider increasing task constraints where possible to shorten the study time and to simplify the task complexity. However, in settings such as conflict resolution (e.g., Findlay & Thagard, 2014) or therapy (Thagard & Larocque, 2020), where capturing causal relationships is crucial, the inclusion of arrows to represent causal influences between events (e.g., event A influences event B) may be advantageous.

This design flexibility allows CAMs to capture a wide range of cognitive representations, yet such complexity might lead to potential issues with task comprehension and instructions and therefore we strongly recommend piloting studies by pre-collecting data from a subset of participants as well as visually inspecting the collected CAMs for clarity and relevance. Piloting ensures alignment between the task demands and the cognitive processes intended to be measured, allowing for necessary adjustments before full-scale data collection.

### Summarizing qualitative data

A central goal for CAM data analysis is to summarize the written text and comments (semantic content) associated with the drawn concepts, which refers to the qualitative analysis part of CAMs. As a possible approach, we developed a *five-step procedure*, which was first introduced by Fenn et al. (2023) and motivated by qualitative research methods (Kuckartz & Rädiker, 2022; Mayring, 2022) as well as existing CAM publications (cf., Livanec et al., 2022; Luthardt et al., 2020; Reuter et al., 2022; Wolfe, 2012). This procedure can be fully implemented using the user interface of the developed data analysis tool, which integrates both automated and manual steps to ensure a thorough and reliable qualitative analysis. The first four steps of the procedure can be handled using the pre-processing functionalities of the data analysis tool (see Table 3), while the final step is supported by the analysis functionalities (see Table 4):

1. *Theoretically driven category identification*: Initially, theoretically relevant superordinate and subordinate categories are identified based on existing literature. It is important to note that identical terms may exhibit positive or negative valence, depending on the argument structure. For example, the term "more free time" might be viewed positively by some and negatively by others during the COVID-19 pandemic.
2. *Visual inspection and memoing*: CAMs are visually inspected and examined individually. Additional superordinate and subordinate categories may emerge during this inspection and are recorded in memos, which capture thoughts, ideas, and hypotheses. The observation process involves systematically inspecting the drawn CAMs, focusing on emerging thematic patterns, to ensure comprehensive identification of categories. Additionally, inspecting CAMs with the lowest and highest mean valence can reveal whether individuals with strong attitudes use different argumentative structures.
3. *Iterative subcategory formation*: Subcategories are for-med iteratively, informed by existing theories. In the initial coding step (3a), categories are generated, and their respective frequencies are documented. In the subsequent coding step (3b), related subcategories within the existing category system are combined, focusing on similar thematic content. This process (3a, 3b) is repeated until all concepts in the CAMs have been coded.
4. *Higher-level topic formation*: All subcategories are combined into topics at a higher level of abstraction.
5. *Results presentation*: The results are presented in the form of tables and graphics.

We recommend to consider concepts’ valence when summarizing CAM data because the valence of a concept reveals how participants emotionally evaluate concepts, which can significantly influence their perceptions and behaviors. For example, the concept of "cost" may be perceived positively when it represents that costs prevent a negatively assessed concept (e.g., high energy consumption), but negatively when it reflects that costs prevent a positively assessed concept (e.g., photovoltaic on houses), making it crucial to account for these emotional nuances in the analysis. Furthermore, it should be explicitly stated how negations and questions were handled. To increase the reliability of categorization with multiple raters, an inter-rater agreement procedure is recommended. Here, it might be helpful to create a coding guideline before categorizing the drawn concepts. Importantly, CAMs could also be quantitatively summarized, for example, by computing network indicators, and these indicators can be related to survey questions or experimental conditions (see “Existing CAM research” section).

We recognize that there are several alternative approaches for summarizing CAM data, including structured, numeric, and content-based analyses. However, we developed the five-step procedure due to its flexibility and applicability within our developed data collection tool, arguing that it effectively incorporates inductive category formation (bottom-up), where categories emerge from the data through iterative coding steps, and deductive category assignment (top-down), where existing theories guide the initial identification of categories (e.g., Kuckartz & Rädiker, 2022). This dual approach ensures a comprehensive, theory-driven analysis while capturing new, participant-specific insights, making it well suited for the often exploratory nature of CAM studies.

## Discussion

The proposed software package C.A.M.E.L. enables researc-hers to collect and analyze belief systems and attitudes within large-scale online studies, as shown in the Appendix B Application example in the Appendix. This method combines both qualitative and quantitative approaches, allowing for comprehensive data collection and analysis. The CAM data sets can be computer-assisted summarized, subsequently analyzed and visualized using multiple implemented functions of the data analysis tool.

We conjecture that CAMs as a mixed-method research tool are very appealing for the following reasons: First, the proposed software package enables large-scale online studies, allowing researchers to gather data from significantly more participants than typically possible in qualitative interview studies. This is because online studies can efficiently collect data from a broader and more diverse sample without the logistical constraints of in-person interviews. Second, because of the free data generation process, participants can indicate novel concepts, that were not yet addressed in the literature and might therefore be missed in regular questionnaire studies.

In the “Guidelines” section and in the online documentation, we provide multiple example studies as a starting point for setting up CAM studies. We encourage researchers to individually adapt the configurations of the data collection tool for their specific needs and to be aware of the influence of possible CAM study designs. As one of several potential approaches for the qualitative analysis of the data, we propose a five-step procedure that allows researchers to apply both an exploratory, bottom-up approach and a theoretically driven method, thereby enabling a comprehensive summarization of the CAM data. By using the data analysis tool, the automatically generated protocol enables full transparency of the summary process of the CAM data. This documentation protocol can be made available together with the data analysis to increase the transparency of the researcher’s decisions and thus supports an open science approach. The functionalities proposed in the analysis part enable the researcher to visualize and report results in an efficient manner. Finally, the administrative panel offers researchers an easy solution to include CAMs in online studies without the need for any coding.

### Reliability & validity

As discussed by Gros et al. (2024), reliability can be measured by comparing CAM network parameters between two measurement time points (test–retest reliability). In their study, CAMs demonstrated good test–retest reliability, with Pearson’s and Spearman’s correlations of up to 0.78 for various network parameters, including mean valence and the number of concepts. This suggests that the CAM method can provide stable measurements across multiple time points, particularly when the topic is associated with stable psychological constructs, such as values (Gros et al., 2024). Additionally, qualitative analysis revealed that 52% of the concepts in the CAMs remained semantically similar between time points, with minimal changes in the underlying thought structure (Gros et al., 2024).

The validity of CAMs has been supported in the first studies. For example, Reuter et al. (2022) and Fenn et al. (2023) emphasized the high face validity of CAMs due to their intuitive, visual representation of complex belief systems. Further, according to Mansell et al. (2021a), preliminary assessments of CAMs involved calculating the likelihood of random replication of CAMs using a Bayesian inference algorithm. The results from this analysis indicated that the CAMs generated in their study reflected intentional decisions by participants, not random assignments. This supports the idea that the structure and content of CAMs are reflective of the participants’ genuine beliefs and emotions, rather than being randomly constructed. Finally, CAMs have demonstrated predictive validity: For example, Mansell et al. (2021b) found significant relationships between CAM network properties (e.g., centrality, density) and participants’ perceptions of risk and political attitudes, providing evidence that CAMs can predict psychological constructs with substantial reliability.

Despite promising findings, particularly when accounting for the inherent complexity of belief systems (see Usó-Doménech & Nescolarde-Selva, 2016), several challenges regarding the reliability and validity of CAMs remain. While test–retest reliability has demonstrated stable measurements over time for a specific topic, further studies exploring additional topics are necessary to support this finding. Although initial studies indicate predictive validity, ongoing research is essential to fully establish the construct validity of CAMs, particularly in identifying the most predictive network parameters and developing new innovative indicators, such as network motifs, as discussed by Levy et al. (2018).

### Conclusion

For the first time here, we present a comprehensive set of interrelated tools for the collection and analysis of CAMs. We think that CAMs could lead to a significantly more realistic assessment of participants’ belief systems and, in turn, probably enhance the predictive capabilities concerning external measures or experimental conditions (cf., Thagard, 2006). Due to their time efficiency, CAMs may prove valuable in addressing various exploratory research questions and could be particularly useful in the early stages of study design, such as informing the development of surveys (see Fenn et al., 2023) or be valuable as a standalone method (Höfele et al., 2022). We hope that the proposed research method of CAMs and the developed tools hold promise in bridging the gap between quantitative and qualitative research.

## Open practices statement

All materials, including source code, are openly available to support transparency and reproducibility. The developed software is hosted on GitHub at https://github.com/CAM-E-L and released under the MIT License, which permits reuse, modification, and distribution. A detailed overview and documentation of the software can be found at https://drawyourminds.de/. All analyses shown in the "Application Example" section can be fully reproduced using the files provided on the Open Science Framework: https://doi.org/10.17605/OSF.IO/P6XSC. The analyses were not preregistered, as they are intended solely for illustrative purposes.

## Figures and Tables

**Fig. 9:**
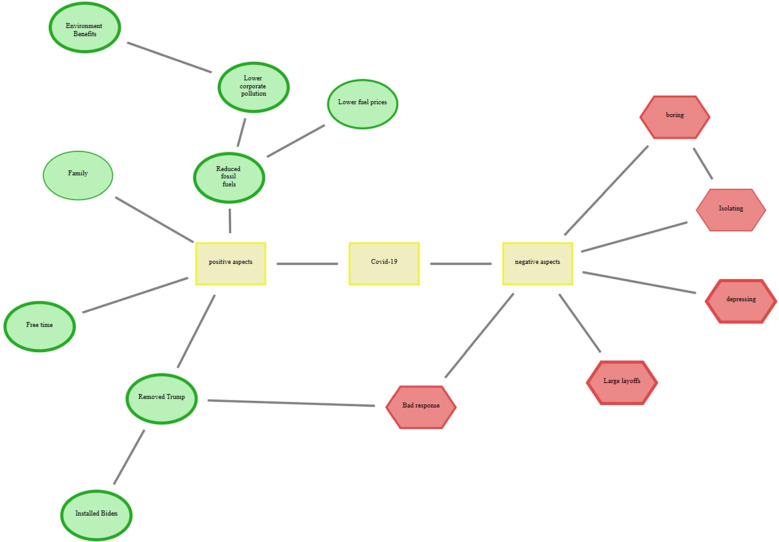
CAM with a high average valence of .63. *Note*. Comment of participant regarding drawn CAM: “there were some negative aspects to COVID-19 but some of the benefits from certain corporations not polluting”

**Fig. 10:**
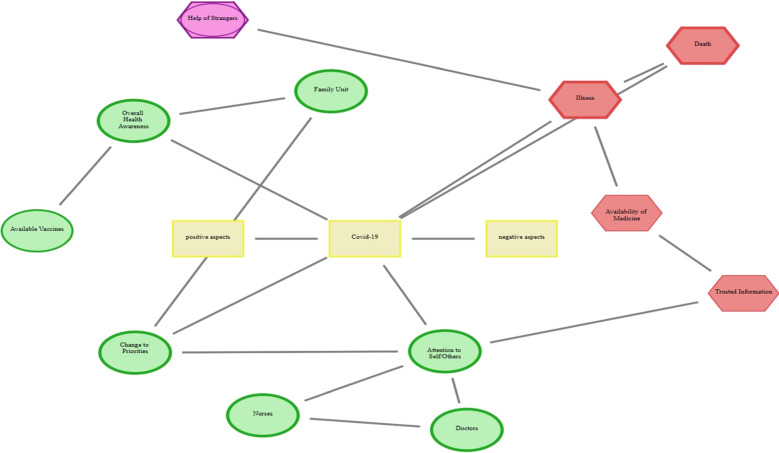
CAM with a high average valence of .80. *Note*. Comment of participant regarding drawn CAM: “I believe that COVID-19 created an awareness of important values and the family unit that would never have been possible without such a horrible pandemic happening and scaring everyone”

**Fig. 11:**
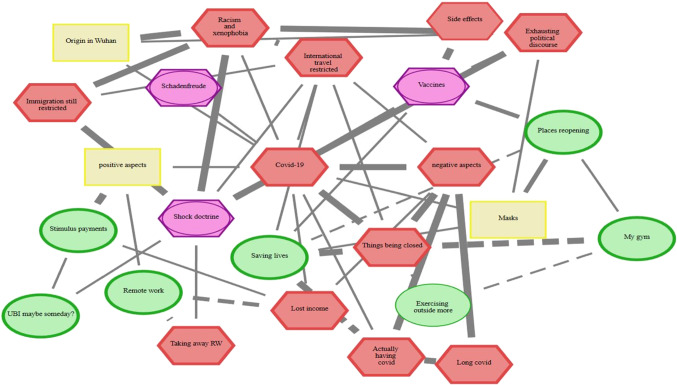
CAM with a low average valence of -.64. *Note*. Comment of participant regarding drawn CAM: “COVID-19 is literally an infectious disease that kills people, why would I feel positively about that?”

**Fig. 12:**
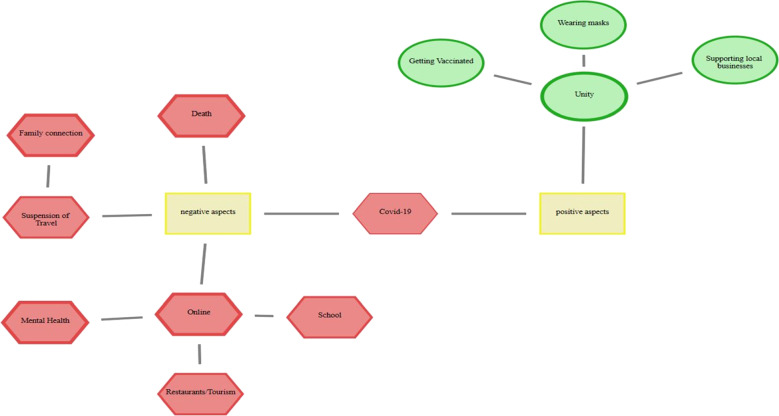
CAM with a low average valence of -.71. *Note*. Comment of participant regarding drawn CAM: “This is as I believe overall COVID-19 was a negative series of events. It is a pandemic after all, and regardless of the unity that people felt to band together, at the end of the day millions were left dead”

**Fig. 14:**
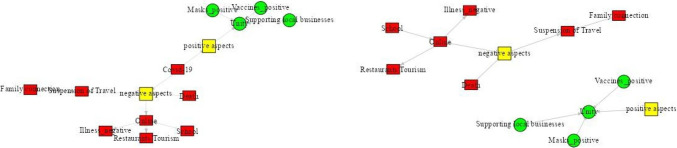
Example of a sliced CAM (Fig. 12). *Note*. The concept “COVID-19” is temporarily removed from the total CAM dataset

## Data Availability

The code for the software can be found on GitHub at https://github.com/CAM-E-L and all the files for the Application Example section on OSF at https://doi.org/10.17605/OSF.IO/P6XSC

## Appendix A Technical details, CAM parameters

### Administrative panel

The administrative panel is a dynamic web page, written in TypeScript within the Next.js web development framework, which enables server-side rendering to increase performance (e.g., Tazetdinov, 2023). The domain is registered by the German Internet service provider Ionos and the Hypertext Transfer Protocol Secure (HTTPS) is used for encryption to secure communication. The server and all the data are stored on Supabase, which is an open source Firebase (cloud computing services by Google) alternative. The frontend (everything the user sees) is separated from the REST API (backend) and it is possible to use the Command Line Interface to interact with the API. When registering, there is an authentication process using the proposed Internet standard JSON Web Token provided by Supabase.

**Table 5:** Selection of most important parameters for the CAM and concept JavaScript classes

Class	Parameter	Meaning	Application
CAM	idCAM	Random character string (ID) that is assigned by the C.A.M.E.L. software to the CAM.	Unique identifier.
	creator	Character string that is stored by the researcher.	Unique ID to identify participants between study parts.
	defocusCAM	Array which stores defocus events, when #fullScreen is set to TRUE	Check if a participant left fullscreen during the CAM study part.
	date	Date of CAM initialization.	Starting point of drawing the CAM.
	nodes	Array which stores all concepts.	Array includes visible and deleted concepts.
	connectors	Array which stores all connectors.	Array includes visible and deleted connectors.
Concept	id	Random character string that is assigned by the C.A.M.E.L. software for each drawn concept.	Unique identifier.
	value	Affective rating given by the participant ranging from [-3, 3].	To compute the average valence of a CAM.
	text	Text written by the participant.	Get meaning of drawn concept.
	comment	Comment given by the participant.	Support interpretation of drawn concept.
	position	{x:,y:} coordinate of the concept.	To compute the distance between concepts.
	isActive	TRUE statement if concept was not deleted.	All deleted concepts are not visible and marked by a FALSE statement.
	date	Date of creation.	Trace the sequence of the drawing process.
	isDraggable	If concept is moveable.	Defined by researcher in advance.
	isDeletable	If concept is deletable.	Defined by researcher in advance.
	isTextChangeable	If text of concept is changeable.	Defined by researcher in advance.
	eventLog	Every interaction with the concept is recorded.	E.g., to create animated videos of drawing process.

*Note*. For detailed information regarding all parameters, please read the "Cognitive-affective map extended logic" section in the online documentation

### Data collection tool

The data collection tool is a static web page. The content that is transmitted to the user’s web browser is exactly as it is stored, typically in the form of plain HTML documents (Nixon, 2021). The visible content of the CAM is within a container for Scalable Vector Graphics (frontend). The CAM itself, as well as its concepts and connectors, are JavaScript classes, which define the structure for these objects (backend): Whenever a participant draws a CAM using the data collection tool, a CAM object is temporarily stored on the client-side. Thereby, the CAM object is a constructor, which is a special type of function used to create and initialize JavaScript objects. Within the CAM object in the "nodes array" and "connectors array", all the created (or deleted) concepts (nodes) and their corresponding connectors (edges) are stored. Thereby, all drawn nodes and connectors are themselves JavaScript classes, which respect classical data model of networks and can thereby be analyzed in real-time using JavaScript libraries like Cytoscape.js^11^ (see "Adaptive designs" example in Table 1). Table 5 contains a selection of the most important parameters for the CAM and concept JavaScript classes.

Further, the class definition respects the classical data model of networks, as such all kinds of real-time pre- or post-processing analyses are possible. Applying the Cytoscape.js library includes a check if participants have drawn two disconnected networks using the breadth-first search algorithm (Kozen, 1992). The configuration of the CAM study, including any predefined concepts as default as a starting point, is defined within a configuration file, which can be hard-coded or adjusted using the administrative panel (no modification possible once the experiment is deployed) or adjusted at the beginning of each single experiment via URL parameters. Most of the user interactions and dialog pop-ups are programmed using the JavaScript library jQuery (Bibeault et al., 2015), which is compatible with all commonly used browsers. To program the tool, we followed classical principles of web development, e.g., differentiating between frontend and backend and using modular programming to make the tool easily adjustable in the future.

### Data analysis tool

As explained in the “Data analysis tool” section, the Shiny application is programmed modularly, which means that single components of the application are divided into separate, independent pieces. Each module consists of a server function, which includes the programming code and a user interface function, which defines the interface of the respective module (Wickham, 2021). By defining global variables, it is possible, for example, to pass the protocol between each module (see concept of scoping in Wickham, 2019). The source code of the data analysis tool can be downloaded via GitHub. Due to its modular programming, additional functionalities can be added relatively easily (for more details, see the online documentation).

## Appendix B Application example

For this application case, we have chosen the study design of a single-time elicitation (see Table 1), whereby participants can freely contribute their thoughts and feelings in addition to predefined concepts, which allows us to gain new insights. We surveyed the participants to reflect on their feelings during the COVID-19 pandemic. We predefined the three concepts "COVID-19", "positive aspects", "negative aspects", whereby "COVID-19" was in the center. For participants, it was not possible to change, move, or delete the predefined concepts.

### Data collection

The online CAM study^12^ was conducted in August 2023 using the participant platform Prolific. The prerequisite for participation was to live in the United States and to speak English fluently. In total, data of ten participants living in the United States were collected, whereby for illustrative purposes we only present the CAM data of four participants in the next section and omit a detailed sample description. The study file, the raw data, and the protocol as well as the outputs of the data analysis tool can be found on OSF: https://doi.org/10.17605/OSF.IO/P6XSC

### Data analysis

We uploaded the collected CAM data to the data analysis tool and drew all CAMs, which enabled us to visually inspect the CAMs and check for possible outliers. Importantly in the notes of the drawn CAMs (see Fig. 9, Figures 10, 11, 12), the feedback of the participants regarding their overall positive or negative affective evaluation is shown. The question regarding their affective evaluation was dynamically generated after participants drew their CAMs in part III (see Fig. 7) and represents a possibility for an "adaptive design" (see Table 1). Using the "Get Report" module, we generated central descriptive statistics, and the four participants drew, for example, on average 17.5 (*SD* = 5.07) concepts (37% were positive, 41% negative, 16% neutral and 6% ambivalent) and the mean average valence over all the CAMs was 0.02 (*SD* = 0.81).

After inspecting each CAM, we summarized the CAM data using the pre-processing part of the data analysis tool, whereby in this case the 60 raw unique concepts (70 in total) were summarized to 55 concepts using four times the "searching terms" and two times the "apply word2vec model" functionalities. After the data were summarized, we used the analysis part of the data analysis tool. Using the module to aggregate CAMs, the two most positive CAMs (see Fig. 9 and 10) were aggregated to Fig. 13 (please refer to Table 4 for the different possibilities to aggregate CAM data). We see that both participants in the online study referred to "Hospital Staff" and "Family", which were both highly positively rated.

Finally, in Fig. 14 the functionality of the "slice CAMs" module is exemplified, whereby the function tries to separate all the CAMs by temporarily removing the central concept "COVID-19". This results in two sub-CAMs (see right side of Fig. 14), which could be separately summarized and analyzed.

It would, however, be easily possible to scale up the online study and compare the participants’ retrospective on the COVID-19 pandemic to existing CAM datasets (Mansell et al., 2021b; Reuter et al., 2021), mental models literature (e.g., de Ridder et al., 2022; Thagard, 2021), qualitative studies (e.g., Strydhorst & Landrum, 2022), or semantic network analysis (e.g., Luo et al., 2021) regarding this topic. It could also be interesting whether a more positive or negative retrospective on the COVID-19 pandemic or certain drawn concepts (e.g., “long COVID”) correlates with mental health outcomes (cf., Bourmistrova et al., 2022).

## Footnotes

1 See our YouTube Video "Software testing to improve the cognitive-affective map tools": https://www.youtube.com/watch?v=T0pUQfCiX1k

2 For an overview of the CAM research at the University of Freiburg, see: https://www.psychologie.uni-freiburg.de/abteilungen/Allgemeine.Psychologie/research/cam-research

3 Publications using cognitive-affective maps: https://paulthagard.com/links/cognitive-affective-maps/

4 A detailed description of the data analysis tool functionalities is out of the scope of this article and so we kindly request the reader to read the ”Data analysis tool” section in the online documentation, see https://camtools-documentation.readthedocs.io/en/master/Data%20Analysis%20Tool/

5 We recommend the reader to read the documentation of the stringr package and download the associated "cheat sheet" (https://github.com/rstudio/cheatsheets/blob/main/strings.pdf); with the following web page it is possible to test combinations of regular expressions in advance: https://regex101.com/

6 The current implementation of both functions is designed for the English CAM data sets. Researchers working with CAM datasets in other languages can either translate their datasets to English before uploading or develop and integrate appropriate dictionaries and language models. By using large language models like "gpt-4o" it is possible to automatically translate the text and comments of the drawn concepts to English before processing the data, the code is available at https://github.com/CAM-E-L/materials

7 Using this approach, it is possible to compute the cosine similarity between pairs of words of currently 25 languages, see: https://spacy.io/models

8 Here we do not elaborate on different ways to instruct participants. Previous studies used a familiar scenario, such as buying at a farmers’ market, to illustrate CAMs. Researchers can adjust the provided instruction versions based on their specific research questions, available at https://github.com/CAM-E-L/materials

9 Advanced users could easily program their own study using for example the JATOS web server (Lange et al., 2015) to host the data collection tool, which is a static web page (see “Appendix A Technical details, CAM parameters” section). For example, by writing a study separated into multiple components (modular programming), it is even possible to send data between components and set up adaptive study designs (e.g., asking participants individually why they have drawn a specific concept as the most central).

10 URL parameters are types of information transferred to a web page when you open it via its web address. They follow a question mark in the web address and multiple parameters can be added using an ampersand ( &).

## Code availability

The code for this project can be found on GitHub at https://github.com/CAM-E-L.

## Declarations

### Code availability

The code for this project can be found on GitHub at https://github.com/CAM-E-L.
